# Striatal abnormalities in trichotillomania: A multi-site MRI analysis

**DOI:** 10.1016/j.nicl.2017.12.031

**Published:** 2017-12-22

**Authors:** Masanori Isobe, Sarah A. Redden, Nancy J. Keuthen, Dan J. Stein, Christine Lochner, Jon E. Grant, Samuel R. Chamberlain

**Affiliations:** a Department of Psychiatry, University of Cambridge, UK; b Department of Neuropsychiatry, Faculty of Medicine, The University of Tokyo Hospital, Japan; c The Nippon Foundation International Fellowship, Japan; d Department of Psychiatry & Behavioral Neuroscience, University of Chicago, USA; e Department of Psychiatry, Massachusetts General Hospital and Harvard Medical School, USA; f MRC Unit on Anxiety & Stress Disorders, Department of Psychiatry, University of Cape Town, South Africa; g Cambridge and Peterborough NHS Foundation Trust, UK

**Keywords:** Trichotillomania, Impulse, Impulsivity, Compulsivity, MRI, Neuroimaging

## Abstract

Trichotillomania (hair-pulling disorder) is characterized by the repetitive pulling out of one's own hair, and is classified as an Obsessive-Compulsive Related Disorder. Abnormalities of the ventral and dorsal striatum have been implicated in disease models of trichotillomania, based on translational research, but direct evidence is lacking. The aim of this study was to elucidate subcortical morphometric abnormalities, including localized curvature changes, in trichotillomania. De-identified MRI scans were pooled by contacting authors of previous peer-reviewed studies that examined brain structure in adult patients with trichotillomania, following an extensive literature search. Group differences on subcortical volumes of interest were explored (*t*-tests) and localized differences in subcortical structure morphology were quantified using permutation testing. The pooled sample comprised N = 68 individuals with trichotillomania and N = 41 healthy controls. Groups were well-matched in terms of age, gender, and educational levels. Significant volumetric reductions were found in trichotillomania patients versus controls in right amygdala and left putamen. Localized shape deformities were found in bilateral nucleus accumbens, bilateral amygdala, right caudate and right putamen. Structural abnormalities of subcortical regions involved in affect regulation, inhibitory control, and habit generation, play a key role in the pathophysiology of trichotillomania. Trichotillomania may constitute a useful model through which to better understand other compulsive symptoms. These findings may account for why certain medications appear effective for trichotillomania, namely those modulating subcortical dopamine and glutamatergic function. Future work should study the state versus trait nature of these changes, and the impact of treatment.

## Highlights

• Trichotillomania is an Obsessive-Compulsive Related Disorder, whose neurobiological basis is poorly understood.
• The basal ganglia are implicated in the generation of motor habits.
• This study confirms microstructural abnormalities of the striatum in patients with trichotillomania, compared to controls.
• These results support the current neurobiological model of trichotillomania.
• Findings may account for why only certain class of medications appear effective for trichotillomania.

## 1 Introduction

Trichotillomania, also known as hair-pulling disorder, is characterized by the repetitive pulling out of one's own hair, leading to significant functional impairment (APA, 2013). The condition has lifetime prevalence of 0.5–1% based on surveys, yet is often hidden, undiagnosed and untreated (Grant et al., 2016, Woods et al., 2006). Trichotillomania has peak age of onset in adolescence, is more common in women than in men, and is currently classified as an Obsessive-Compulsive Related Disorder (Grant and Chamberlain, 2016). However, in contrast to the repetitive compulsive acts observed in obsessive-compulsive disorder (OCD), repetitive behaviors in trichotillomania are not generally driven by intrusive thoughts. As such, and in view of the recent development of animal models with good validity (Chamberlain et al., 2007b, Greer and Capecchi, 2002, Hyman, 2007), trichotillomania constitutes a key model for better understanding compulsive symptoms more generally. However, surprisingly little is known about the neurobiological basis of this disorder in humans (Christenson et al., 1993, Cohen et al., 1995, Mansueto et al., 2007, Odlaug and Grant, 2010).

Reviewing available clinical and imaging studies of trichotillomania, previous work suggested an “ABC” model of trichotillomania emphasizing the dysfunction of pathways involved in Affect regulation, Behavioral Control, and Cognition (Stein et al., 2006). This approach implicates, in turn, the frontal cortices (serving to regulate impulses and habits), the amygdala (involved in emotional processing) (Canli et al., 2005), and the striatum (playing key roles in reward processing and motor outflow) (Ahmari et al., 2013, Knutson et al., 2001). In keeping with this, studies have found that trichotillomania is associated with impairment on response inhibition tests (Chamberlain et al., 2006, Odlaug et al., 2014), and phenomenological studies have found relationships between emotional states (dysphoria, anxiety) and the severity of the hair-pulling symptoms (Grant et al., 2017).

Neuroimaging constitutes a core modality through which to evaluate implicated neural regions in patients with trichotillomania. Structural imaging studies comparing patients with trichotillomania to controls have yielded mixed results with regards to the basal ganglia. One study found no volumetric changes in the caudate (Stein et al., 1997), one found no difference in the global basal ganglia (Roos et al., 2015), one found reduced left putamen volumes (O'Sullivan et al., 1997), and another found excess grey matter density in left putamen and amygdala (Chamberlain et al., 2008). Due to the relatively limited research scrutiny of this disorder, and limited funding, imaging studies have typically involved relatively small sample sizes. Small sample sizes result in limited statistical power and elevate the risk of false positive findings (Button et al., 2013). Subcortical structures are difficult to visualize due to poor and variable signal intensity (as compared to cortex) (Patenaude et al., 2011) and several mainstream imaging analysis pipelines were designed for analysis of cortex rather than subcortical regions (Dale et al., 1999). More recent pipelines enable the sensitive measurement not only of volumes of subcortical structures, but also of local differences in deformations of shape across groups; the latter has the advantage of not relying on arbitrary smoothing extent or tissue classification (Patenaude et al., 2011).

Therefore, the current study pooled together raw MRI scans from all available peer-reviewed case-control studies of trichotillomania, and evaluated the volume and morphology of select subcortical structures. Software pipelines, including “vertex analysis” from FMRIB's Software Library (FSL) were used, these being designed specifically for the sensitive measurement of subcortical structures (Patenaude et al., 2011). We hypothesized that trichotillomania would be associated with volumetric and morphometric abnormalities of the caudate, putamen, nucleus accumbens, and amygdala (Stein et al., 2006).

## 2 Material and methods

### 2.1 Data collection of participants

Conventional cortical data for the current sample were reported previously and the MRI dataset obtained here was the same as that used by the previous study (Chamberlain et al., 2017). In brief, all structural MRI studies regarding trichotillomania were identified via PubMed in February 2017. We contacted the authors of these publications and invited them to contribute de-identified MRI scans from published studies, subject to original participants providing appropriate consent and Institutional Board Approvals. De-identified T1-weighted MRI images and demographic data were shared for patients and controls. Demographic data consisted of age, gender, level of education, medication status, and severity of illness measured with the Massachusetts General Hospital Hair Pulling Scale (MGH-HPS) (Keuthen et al., 2007), which is a self-administered questionnaire assessing severity of trichotillomania. We excluded trichotillomania patients who were taking psychotropic medication at the time of study participation, to avoid potentially confounding effects of medication on brain structure (McDonald, 2015). This applied to six patients.

### 2.2 Data analysis

Group differences in demographic data were explored with independent sample *t*-tests (p < 0.05, two-tailed, uncorrected) and chi-square tests (p < 0.05), using JMP Pro.

Imaging pre-processing and data extractions were undertaken on the University of Chicago Midway computing system. The T1-weighted images of each subject were preprocessed. They were automatically bias-field corrected and non-linearly registered to the MNI 152 standard space. We employed FMRIB's Integrated Registration and Segmentation Tool (FIRST) implemented in FSL 5.0.9 to automatically segment subcortical structures (Patenaude et al., 2011). Segmentation was based on shape models with structural boundaries obtained from 336 manually segmented images, and resulted in a deformable surface mesh of each subcortical structure consisting of vertices. The meshes were reconstructed and filled in MNI space and boundary correction was applied. Then, the segmented images were transformed into original space. All segmented images were visually checked for errors in registration and segmentation and the images of 2 trichotillomania patients were discarded due to poor quality in segmentation.

#### 2.2.1 Volumetric analysis

Subcortical volumes of the bilateral nucleus accumbens, amygdala, caudate, and putamen were extracted. These regions of interest were selected based on extant models of the pathophysiology of trichotillomania (Stein et al., 2006). We calculated total intracranial volume (ICV) as the sum volumes of grey matter, white matter and cerebrospinal fluid using FMRIB's Automated Segmentation Tool (FAST) (Zhang et al., 2001). Each subject's brain scan was skull-stripped with the Brain Extraction Tool and linearly aligned to the MNI152 space, and the inverse of the determinant of the affine transformation matrix computed by the software was multiplied by the ICV size of the template. We adjusted the subcortical volumes by the ICV of each patient (Buckner et al., 2004). The adjusted volumes of each participant were exported into JMP Pro Version 13.1.0. Group differences in ICV-corrected subcortical volumes were explored using independent sample *t*-tests. Statistical significance was defined as p < 0.05 two-tailed, Bonferroni corrected. Correlations between MGH-HPS scores and subcortical volumes were analyzed in trichotillomania participants, using Spearman's rho. For correlation analyses, significance was defined as p < 0.05 two-tailed uncorrected.

#### 2.2.2 Vertex analysis

Vertex analysis, implemented in FIRST, (FSL), was employed to compare the shapes of the subcortical structures between groups (Patenaude et al., 2011). The vertex locations of each participant were projected onto the surface normal of the average shape template of the 336 training subjects provided by FSL, and the perpendicular distance from the average surface was calculated. Negative value of the vertex represented deformation in the inward direction and positive value of a vertex indicated deformation in the outward direction. These values were compared between groups using ‘Randomise’, a permutation-based non-parametric testing method implemented in FSL with 5000 iterations (Winkler et al., 2014). The statistical images were produced with Threshold-Free Cluster Enhancement (TFCE) for multiple comparisons (Smith and Nichols, 2009), in which threshold was set at p < 0.05.

## 3 Results

### 3.1 Demographics

The final study sample comprised 68 individuals with trichotillomania and 41 healthy controls. The mean total Massachusetts General Hospital Hair Pulling Scale severity score in the trichotillomania group was 15.6 (standard deviation 4.7), consistent with, on average, mild-moderate illness. There were no significant differences in age, gender, education level, total grey and white matter volumes, or total intracranial volume, between the groups (Table 1).

**Table 1:** Comparison of demographics and clinical characteristics in the trichotillomania and healthy control groups. Groups did not differ significantly on these measures (all p > 0.10).

	Trichotillomania	Healthy controls	Statistic	p-Value
	(N = 68)	(N = 41)		
Age (mean, SD)	33.49 (11.78)	32.42 (10.76)	0.48^a^	0.64
Gender (N, %)				
Male	6 (8.8%)	5 (12.2%)	0.32^b^	0.57
Female	62 (91.2%)	36 (87.8%)		
Education (N, %)				
High school or less	6 (8.8%)	5 (12.5%)	0.40^b^	0.82
College/lower degree	15 (22.1%)	9 (22.5%)		
Graduate/higher degree	47 (69.1%)	26 (65.0%)		
Total grey matter volume, mm^3^	571,505 (54517)	565,064 (45568)	0.63^a^	0.53
Total white matter volume, mm^3^	507,156 (49080)	498,500 (43328)	0.93^a^	0.35
Total intracranial volume, mm^3^	1,343,947 (107840)	1,330,783 (103409)	0.63^a^	0.53

Abbreviations: SD = standard deviation.

a Independent sample *t*-tests.

b Chi-square tests.

### 3.2 Volumetric analysis

Absolute volumetric data of subcortical grey matter regions were listed and results of group comparisons are shown in Table 2. With correction for multiple comparisons, patients had significantly smaller ICV-corrected subcortical volumes compared to controls in the right amydala (t = − 2.84, uncorrected p = 0.005, corrected p = 0.04) and in the left putamen (t = − 2.99, uncorrected p = 0.004, corrected p = 0.032). There were trends toward volumetric reductions in other subcortical regions, but these did not withstand correction for multiple comparisons (Table 2). Volumes of subcortical regions did not correlate significantly with symptom severity in the trichotillomania cases (all p > 0.05 uncorrected).

**Table 2:** Comparison of subcortical volumes among individuals with trichotillomania and healthy controls. *p < 0.05, **p < 0.01 significantly smaller volumes in patients compared to controls with Bonferroni correction.

Subcortical structures	*t*-test	Uncorrected p-value	Raw volume of subcortical structures (SD), mm^3^	
			Trichotillomania (N = 68)	Healthy controls (N = 41)
Lt. Amygdala	− 2.31	0.024	1183.97 (293.59)	1308.31 (289.79)
Rt. Amygdala	− 2.84	0.005*	1159.68 (294.21)	1309.58 (272.03)
Lt. Putamen	− 2.99	0.004*	5034.06 (737.89)	5366.68 (664.66)
Rt. Putamen	− 2.71	0.008	4996.79 (597.69)	5222.66 (547.46)
Lt. Caudate	− 1.80	0.075	3458.6 (482.0)	3575.0 (430.0)
Rt. Caudate	− 1.25	0.216	3640.5 (443.9)	3712.2 (429.2)
Lt. Nuc. Accumbens	− 1.91	0.059	553.0 (139.4)	594.0 (109.9)
Rt. Nuc. Accumbens	− 1.75	0.084	434.0 (118.6)	470.8 (115.0)

### 3.3 Vertex analysis

**Fig. 1:**
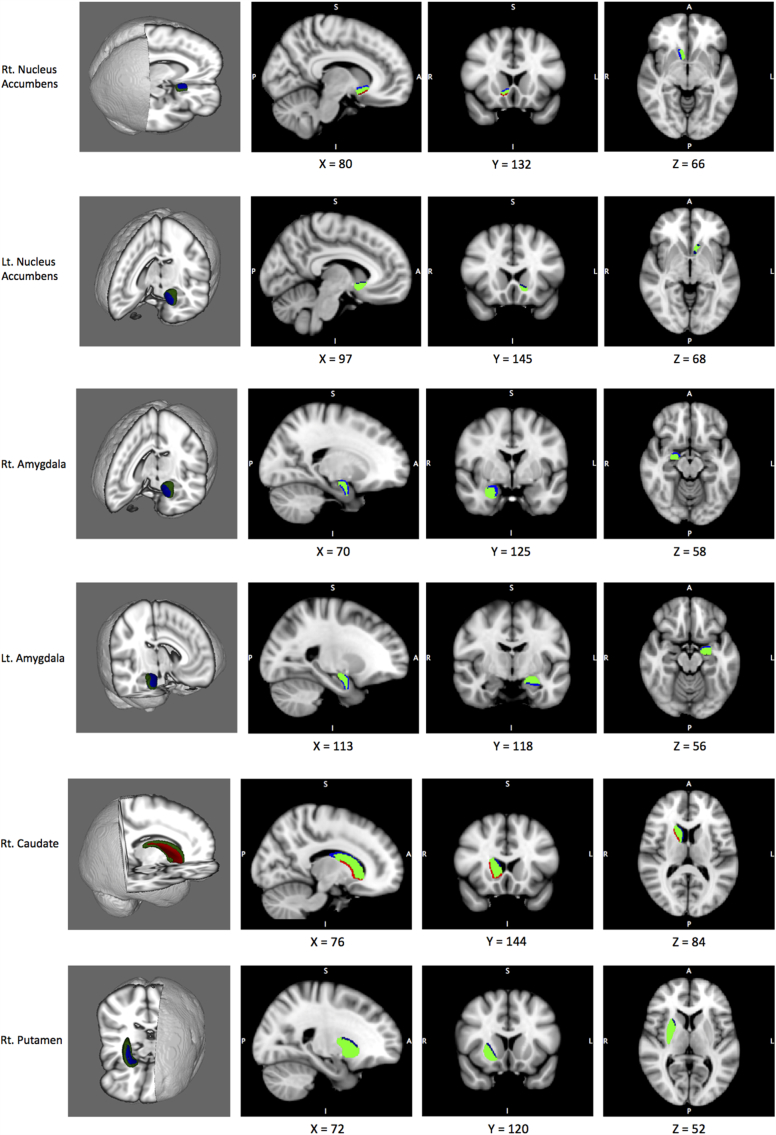
Significant deformations of subcortical structures in patients with trichotillomania compared to controls, at p < 0.05 corrected by permutation tests. In blue, localized surface contractions in patients; in red, localized surface expansions in patients. Template outlines of structures are shown in green.

The results of vertex-wise shape analysis are shown in Fig. 1. The analysis revealed localized shape deformities in trichotillomania patients versus controls in the right putamen, right caudate, bilateral nucleus accumbens, and bilateral amygdala.

## 4 Discussion

This study elucidated subcortical structural characteristics in trichotillomania, using pooled MRI data from available peer-reviewed imaging studies, coupled with statistical pipelines designed to overcome problems in visualizing such non-cortical structures. The main findings were that, compared to matched healthy volunteers, trichotillomania patients had (i) significant volume reductions of the right amygdala and left putamen; and (ii) localized morphometric (curvature) abnormalities of the putamen, caudate, nucleus and amygdala.

The putamen is the key component in motor control and is involved in habit learning and response suppression across species (Morris et al., 2016). While the caudate plays a role in directed-learning (such as during complex planning or high-level flexible learning tasks), the putamen is more involved in lower-level stimulus-response habit learning (Grahn et al., 2008). The macroscopic changes in putamen but not caudate volume associated with trichotillomania may help to account for why co-morbidity free patients with trichotillomania manifest response inhibition deficits (involving simple stimulus-response mappings (Odlaug et al., 2014)) while other cognitive domains are relatively spared (e.g. executive planning, set-shifting (Chamberlain et al., 2007a)). We suggest that the more pronounced changes in the putamen are also in keeping with trichotillomania being a disorder of motor habit, rather than the symptoms being primarily driven by cognitions or more complex sequencing (Grahn et al., 2008). Abnormalities of the putamen have also been implicated in tic spectrum disorders including Tourette's syndrome (Singer et al., 1993). In a recent study of patients with tics, un-medicated patients relied on not goal-directed but rather on habitual behavioral control, and showed stronger structural connectivity between the supplementary motor cortex and putamen (Delorme et al., 2016). Healthy volunteer studies support a role for dopamine D2 receptors in the putamen in regulating response inhibition (Ghahremani et al., 2012), which may in turn support a role for dorsal striatum dopamine receptors in the pathophysiology of trichotillomania, with potential treatment implications.

In addition to macroscopic volume changes in the left putamen, we also found here that the right amygdala was abnormally small in patients with trichotillomania compared to healthy controls. The amygdala was traditionally held to be important in fear-processing (Ohman and Mineka, 2001), but more recent work highlights its involvement in a broader range of processes, including arousal, attention, value representation and decision-making (Koen et al., 2016, Pessoa, 2010). Negative affective states can directly contribute to and trigger hair-pulling symptoms (Christenson et al., 1993), and individuals with trichotillomania showed decreased functional connectivity of amygdala within the reward network (White et al., 2013). It has also been suggested that hair-pulling may serve to regulate arousal levels: for some individuals, hair-pulling may be undertaken during times of boredom or low activity (e.g. watching television); while for others, hair-pulling may be soothing and undertaken during times of stress and arousal (e.g. work stress (Stein et al., 2006)).

Using an imaging pipeline that generated a mesh, representing the curvature (three-dimensional morphology) of subcortical structures, there was evidence for localized abnormalities in the curvature of the amygdala, putamen, caudate, and nucleus accumbens, in trichotillomania patients compared to the controls. This is the first study to examine localized structural changes in this disorder. These findings should be viewed as being more subtle than the macroscopic differences described above, and thus warrant replication in future work. Interestingly, the nucleus accumbens plays a role in impulsivity (Dalley et al., 2011) including in temporal reward discounting (Cardinal et al., 2001) and premature responding (Christakou et al., 2004). In a previous functional neuroimaging study, individuals with trichotillomania showed decreased activation of nucleus accumbens during reward anticipation (White et al., 2013). It has also been suggested that caudate and the subthalamic nucleus have important roles in response inhibition performance (Eagle and Robbins, 2003), as well as the putamen.

Several limitations in the present study should be considered. First, demographic and clinical data were limited to a few key measures, because the study pooled data from different research programs. It would be valuable to explore relationships between the structural abnormalities found here and other measures (such as cognitive functioning, and questionnaires pertaining to affect dysregulation). Second, the current study did not evaluate any effects of psychotropic medication on brain structure in trichotillomania – we excluded patients taking medications. There is no widely accepted pharmacological ‘standard of care’ for this disorder (Rothbart et al., 2013), which means that evaluation of medication effects would likely be a moot point in the absence of large data using evidence-based pharmacological treatments. Third, the findings here differ from those we reported previously in an overlapping sample (Chamberlain et al., 2017). In this previous work, we focused on cortical structure but also reported select subcortical volumes extracted using the Freesurfer pipeline, and group differences were not significant. The current study used the FIRST algorithm, which appears superior to Freesurfer for analysis of at least some subcortical structures (notably putamen) in terms of accuracy (Perlaki et al., 2017). Thus viewed together, the findings suggest that FIRST may be more sensitive to subcortical changes in trichotillomania. It is also interesting to note that the variability (standard deviations) were somewhat larger in the Freesurfer study for subcortical regions, which may again suggest lower precision for this alternative imaging pipeline as pertains to subcortical structures. Finally, by pooling scans from multiple sites, we were able to achieve a larger sample size; however, the sample size was still relatively small compared to mega-analyses available for other related disorders (e.g. OCD) (Boedhoe et al., 2017).

## 5 Conclusion

In summary, this analysis found subcortical brain abnormalities in trichotillomania: reduced putamen and amygdala volumes coupled with more subtle localized changes in the curvature of the putamen, caudate, accumbens, and amygdala. Thus, abnormalities of neural nodes mediating affect regulation, reward-processing, and habit generation, all appear to be involved in the pathophysiology of trichotillomania. Future work should examine the temporal course of these changes and their genetic underpinnings (potential heritability): it may be that some changes constitute vulnerability markers but others are secondary to the symptoms (or reflect compensatory changes during brain development). From a treatment perspective, it is interesting that *n*-acetyl cysteine (an amino-acid precursor), a glutamatergic medication, has efficacy in the treatment of trichotillomania. *N*-acetyl cysteine is an amino acid precursor that restores extracellular levels of glutamate in the nucleus accumbens in animal models of substance use. Given the role of D2 dopamine receptors in mediating response inhibition in the striatum (Ghahremani et al., 2012), and beneficial effects seen in trichotillomania with olanzapine, the current results may hint at why only certain medications appear to have efficacy in treating this neglected disorder.

## Compliance with ethical standards

The research complied with relevant ethical standards including the Declaration of Helsinki.

## Funding

This work was funded by a Wellcome Trust Clinical Fellowship to Dr. Chamberlain (UK; Reference 110049/Z/15/Z) and by a grant from the Trichotillomania Learning Center to Dr. Grant. Drs. Lochner and Stein were funded by the South African Medical Research Council. Dr. Keuthen was funded by an anonymous benefactor for the collection of her imaging data. Dr. Isobe's role in this project was funded by Grant-in-Aid for Scientific Research on Innovative Areas (16K21720) from the Ministry of Education, Culture, Sports, Science and Technology of Japan (MEXT) and by the Nippon Foundation International Fellowship.

## Potential conflicts of interest

Dr. Chamberlain consults for Cambridge Cognition and Shire. Dr. Grant is chair of the Scientific Advisory Board of the TLC Foundation for BFRBs and currently receives funding from its BFRB Precision Medicine Initiative. In addition, he has received research grants from NIDA, American Foundation for Suicide Prevention, National Center for Responsible Gaming, Brainsway, and Takeda Pharmaceuticals. He receives yearly compensation from Springer Publishing for acting as Editor-in-Chief of the Journal of Gambling Studies and has received royalties from Oxford University Press, American Psychiatric Publishing, Inc., Norton Press, and McGraw Hill. Dr. Keuthen is on the Scientific Advisory Board of the TLC Foundation for BFRBs and currently receives funding from its BFRB Precision Medicine Initiative. She receives royalties from New Harbinger, Inc. In the past 3 years, Dr. Stein has received research grants and/or consultancy honoraria from Biocodex, Lundbeck, Servier, and Sun. The other authors report no potential conflicts of interest.

## Ethical approval

Research studies were approved by ethics committee.

## Informed consent

Participants provided informed consent.

## References

[bb0005] Ahmari S.E., Spellman T., Douglass N.L., Kheirbek M.A., Simpson H.B., Deisseroth K., Gordon J.A., Hen R. (2013). Repeated cortico-striatal stimulation generates persistent OCD-like behavior. Science.

[bb0010] APA (2013). Diagnostic and Statistical Manual of Mental Disorders: DSM-5.

[bb0015] Boedhoe P.S., Schmaal L., Abe Y., Ameis S.H., Arnold P.D., Batistuzzo M.C., Benedetti F., Beucke J.C., Bollettini I., Bose A., Brem S., Calvo A., Cheng Y., Cho K.I., Dallaspezia S., Denys D., Fitzgerald K.D., Fouche J.P., Gimenez M., Gruner P., Hanna G.L., Hibar D.P., Hoexter M.Q., Hu H., Huyser C., Ikari K., Jahanshad N., Kathmann N., Kaufmann C., Koch K., Kwon J.S., Lazaro L., Liu Y., Lochner C., Marsh R., Martinez-Zalacain I., Mataix-Cols D., Menchon J.M., Minuzzi L., Nakamae T., Nakao T., Narayanaswamy J.C., Piras F., Piras F., Pittenger C., Reddy Y.C., Sato J.R., Simpson H.B., Soreni N., Soriano-Mas C., Spalletta G., Stevens M.C., Szeszko P.R., Tolin D.F., Venkatasubramanian G., Walitza S., Wang Z., van Wingen G.A., Xu J., Xu X., Yun J.Y., Zhao Q., Thompson P.M., Stein D.J., van den Heuvel O.A. (2017). Distinct subcortical volume alterations in pediatric and adult OCD: a worldwide meta- and mega-analysis. Am. J. Psychiatry.

[bb0020] Buckner R.L., Head D., Parker J., Fotenos A.F., Marcus D., Morris J.C., Snyder A.Z. (2004). A unified approach for morphometric and functional data analysis in young, old, and demented adults using automated atlas-based head size normalization: reliability and validation against manual measurement of total intracranial volume. NeuroImage.

[bb0025] Button K.S., Ioannidis J.P., Mokrysz C., Nosek B.A., Flint J., Robinson E.S., Munafo M.R. (2013). Power failure: why small sample size undermines the reliability of neuroscience. Nat. Rev. Neurosci..

[bb0030] Canli T., Omura K., Haas B.W., Fallgatter A., Constable R.T., Lesch K.P. (2005). Beyond affect: a role for genetic variation of the serotonin transporter in neural activation during a cognitive attention task. Proc. Natl. Acad. Sci. U. S. A..

[bb0035] Cardinal R.N., Pennicott D.R., Sugathapala C.L., Robbins T.W., Everitt B.J. (2001). Impulsive choice induced in rats by lesions of the nucleus accumbens core. Science.

[bb0040] Chamberlain S.R., Fineberg N.A., Blackwell A.D., Robbins T.W., Sahakian B.J. (2006). Motor inhibition and cognitive flexibility in obsessive-compulsive disorder and trichotillomania. Am. J. Psychiatry.

[bb0045] Chamberlain S.R., Fineberg N.A., Blackwell A.D., Clark L., Robbins T.W., Sahakian B.J. (2007). A neuropsychological comparison of obsessive-compulsive disorder and trichotillomania. Neuropsychologia.

[bb0050] Chamberlain S.R., Menzies L., Sahakian B.J., Fineberg N.A. (2007). Lifting the veil on trichotillomania. Am. J. Psychiatry.

[bb0055] Chamberlain S.R., Menzies L.A., Fineberg N.A., Del Campo N., Suckling J., Craig K., Muller U., Robbins T.W., Bullmore E.T., Sahakian B.J. (2008). Grey matter abnormalities in trichotillomania: morphometric magnetic resonance imaging study. Br. J. Psychiatry.

[bb0060] Chamberlain S.R., Harries M., Redden S.A., Keuthen N.J., Stein D.J., Lochner C., Grant J.E. (2017). Cortical thickness abnormalities in trichotillomania: international multi-site analysis. Brain Imaging Behav..

[bb0065] Christakou A., Robbins T.W., Everitt B.J. (2004). Prefrontal cortical-ventral striatal interactions involved in affective modulation of attentional performance: implications for corticostriatal circuit function. J. Neurosci..

[bb0070] Christenson G.A., Ristvedt S.L., Mackenzie T.B. (1993). Identification of trichotillomania cue profiles. Behav. Res. Ther..

[bb0075] Cohen L.J., Stein D.J., Simeon D., Spadaccini E., Rosen J., Aronowitz B., Hollander E. (1995). Clinical profile, comorbidity, and treatment history in 123 hair pullers: a survey study. J. Clin. Psychiatry.

[bb0080] Dale A.M., Fischl B., Sereno M.I. (1999). Cortical surface-based analysis. I. Segmentation and surface reconstruction. NeuroImage.

[bb0085] Dalley J.W., Everitt B.J., Robbins T.W. (2011). Impulsivity, compulsivity, and top-down cognitive control. Neuron.

[bb0090] Delorme C., Salvador A., Valabregue R., Roze E., Palminteri S., Vidailhet M., de Wit S., Robbins T., Hartmann A., Worbe Y. (2016). Enhanced habit formation in Gilles de la Tourette syndrome. Brain.

[bb0095] Eagle D.M., Robbins T.W. (2003). Inhibitory control in rats performing a stop-signal reaction-time task: effects of lesions of the medial striatum and d-amphetamine. Behav. Neurosci..

[bb0100] Ghahremani D.G., Lee B., Robertson C.L., Tabibnia G., Morgan A.T., De Shetler N., Brown A.K., Monterosso J.R., Aron A.R., Mandelkern M.A., Poldrack R.A., London E.D. (2012). Striatal dopamine D(2)/D(3) receptors mediate response inhibition and related activity in frontostriatal neural circuitry in humans. J. Neurosci..

[bb0105] Grahn J.A., Parkinson J.A., Owen A.M. (2008). The cognitive functions of the caudate nucleus. Prog. Neurobiol..

[bb0110] Grant J.E., Chamberlain S.R. (2016). Trichotillomania. Am. J. Psychiatry.

[bb0115] Grant J.E., Redden S.A., Leppink E.W., Odlaug B.L., Chamberlain S.R. (2016). Psychosocial dysfunction associated with skin picking disorder and trichotillomania. Psychiatry Res..

[bb0120] Grant J.E., Redden S.A., Medeiros G.C., Odlaug B.L., Curley E.E., Tavares H., Keuthen N.J. (2017). Trichotillomania and its clinical relationship to depression and anxiety. Int. J. Psychiatry Clin. Pract..

[bb0125] Greer J.M., Capecchi M.R. (2002). Hoxb8 is required for normal grooming behavior in mice. Neuron.

[bb0130] Hyman S.E. (2007). Neuroscience: obsessed with grooming. Nature.

[bb0135] Keuthen N.J., Flessner C.A., Woods D.W., Franklin M.E., Stein D.J., Cashin S.E., Trichotillomania Learning Center Scientific Advisory, B (2007). Factor analysis of the Massachusetts General Hospital Hairpulling Scale. J. Psychosom. Res..

[bb0140] Knutson B., Fong G.W., Adams C.M., Varner J.L., Hommer D. (2001). Dissociation of reward anticipation and outcome with event-related fMRI. Neuroreport.

[bb0145] Koen N., Fourie J., Terburg D., Stoop R., Morgan B., Stein D.J., van Honk J. (2016). Translational neuroscience of basolateral amygdala lesions: studies of Urbach-Wiethe disease. J. Neurosci. Res..

[bb0150] Mansueto C.S., Thomas A.M., Brice A.L. (2007). Hair pulling and its affective correlates in an African-American university sample. J. Anxiety Disord..

[bb0155] McDonald C. (2015). Brain structural effects of psychopharmacological treatment in bipolar disorder. Curr. Neuropharmacol..

[bb0160] Morris L.S., Kundu P., Dowell N., Mechelmans D.J., Favre P., Irvine M.A., Robbins T.W., Daw N., Bullmore E.T., Harrison N.A., Voon V. (2016). Fronto-striatal organization: defining functional and microstructural substrates of behavioural flexibility. Cortex.

[bb0165] Odlaug B.L., Grant J.E. (2010). Impulse-control disorders in a college sample: results from the self-administered Minnesota Impulse Disorders Interview (MIDI). Prim. Care Companion J. Clin. Psychiatry.

[bb0170] Odlaug B.L., Chamberlain S.R., Derbyshire K.L., Leppink E.W., Grant J.E. (2014). Impaired response inhibition and excess cortical thickness as candidate endophenotypes for trichotillomania. J. Psychiatr. Res..

[bb0175] Ohman A., Mineka S. (2001). Fears, phobias, and preparedness: toward an evolved module of fear and fear learning. Psychol. Rev..

[bb0180] O'Sullivan R.L., Rauch S.L., Breiter H.C., Grachev I.D., Baer L., Kennedy D.N., Keuthen N.J., Savage C.R., Manzo P.A., Caviness V.S., Jenike M.A. (1997). Reduced basal ganglia volumes in trichotillomania measured via morphometric magnetic resonance imaging. Biol. Psychiatry.

[bb0185] Patenaude B., Smith S.M., Kennedy D.N., Jenkinson M. (2011). A Bayesian model of shape and appearance for subcortical brain segmentation. NeuroImage.

[bb0190] Perlaki G., Horvath R., Nagy S.A., Bogner P., Doczi T., Janszky J., Orsi G. (2017). Comparison of accuracy between FSL's FIRST and Freesurfer for caudate nucleus and putamen segmentation. Sci. Rep. 2017 May 25.

[bb0195] Pessoa L. (2010). Emotion and cognition and the amygdala: from “what is it?” to “what's to be done?”. Neuropsychologia.

[bb0200] Roos A., Grant J.E., Fouche J.P., Stein D.J., Lochner C. (2015). A comparison of brain volume and cortical thickness in excoriation (skin picking) disorder and trichotillomania (hair pulling disorder) in women. Behav. Brain Res..

[bb0205] Rothbart R., Amos T., Siegfried N., Ipser J.C., Fineberg N., Chamberlain S.R., Stein D.J. (2013). Pharmacotherapy for trichotillomania. The Cochrane Database of Systematic Reviews.

[bb0210] Singer H.S., Reiss A.L., Brown J.E., Aylward E.H., Shih B., Chee E., Harris E.L., Reader M.J., Chase G.A., Bryan R.N. (1993). Volumetric MRI changes in basal ganglia of children with Tourette's syndrome. Neurology.

[bb0215] Smith S.M., Nichols T.E. (2009). Threshold-free cluster enhancement: addressing problems of smoothing, threshold dependence and localisation in cluster inference. NeuroImage.

[bb0220] Stein D.J., Wessels C., Carr J., Hawkridge S., Bouwer C., Kalis N. (1997). Hair pulling in a patient with Sydenham's chorea. Am. J. Psychiatry.

[bb0225] Stein D.J., Chamberlain S.R., Fineberg N. (2006). An A-B-C model of habit disorders: hair-pulling, skin-picking, and other stereotypic conditions. CNS Spectr..

[bb0230] White M.P., Shirer W.R., Molfino M.J., Tenison C., Damoiseaux J.S., Greicius M.D. (2013). Disordered reward processing and functional connectivity in trichotillomania: a pilot study. J. Psychiatr. Res..

[bb0235] Winkler A.M., Ridgway G.R., Webster M.A., Smith S.M., Nichols T.E. (2014). Permutation inference for the general linear model. NeuroImage.

[bb0240] Woods D.W., Flessner C.A., Franklin M.E., Keuthen N.J., Goodwin R.D., Stein D.J., Walther M.R. (2006). The Trichotillomania Impact Project (TIP): exploring phenomenology, functional impairment, and treatment utilization. J. Clin. Psychiatry.

[bb0245] Zhang Y., Brady M., Smith S. (2001). Segmentation of brain MR images through a hidden Markov random field model and the expectation-maximization algorithm. IEEE Trans. Med. Imaging.

